# Does robotic circumferential oversewing reduce anastomotic leakage in stapled anastomosis for rectal cancer surgery?

**DOI:** 10.1007/s10151-025-03207-2

**Published:** 2025-08-14

**Authors:** J.-M. Jung, S. Yang, Y. S. Yoon, Y. I. Kim, M. H. Kim, J. L. Lee, C. W. Kim, I. J. Park, S.-B. Lim, C. S. Yu

**Affiliations:** 1 Department of Surgery, Ajou University School of Medicine, Suwon, South Korea; 2 Department of Surgery, Ulsan University Hospital, University of Ulsan College of Medicine, Ulsan, South Korea; 3 Division of Colon and Rectal Surgery, Department of Surgery, Asan Medical Center, University of Ulsan College of Medicine, Seoul, South Korea

**Keywords:** Rectal neoplasms, Anastomotic leak, Robot surgical procedures, Suture techniques

## Abstract

**Background:**

Anastomotic leakage (AL) remains a challenging complication of rectal cancer surgery. In patients diagnosed with low risk of AL, low anterior resection (LAR) is often performed without creating a stoma. However, AL can still occur even in patients considered to be at low risk. This study assessed the effects of circumferential oversewing (CO) on AL in patients undergoing robotic LAR without fecal diversion.

**Methods:**

We retrospectively reviewed data from 225 patients with rectal cancer who underwent robotic LAR without fecal diversion. They were divided into CO and non-CO groups. The CO group received oversewing along the circular staple line. The AL rate was assessed after the inverse probability of treatment weighting (IPTW) adjustments.

**Results:**

After IPTW adjustment, no significant differences in baseline characteristics were observed between the two groups. Overall complication and AL rates were 12.0% and 4.5%, respectively. Although no difference in overall complications was observed between the two groups, patients in the CO group had a significantly lower AL rate than the non-CO group (1.7% vs. 10.3%, *p* = 0.010). Logistic regression analysis revealed that the CO procedure was a protective factor against AL (IPTW-adjusted OR 0.153, 95% CI 0.036–0.643, *p* = 0.010).

**Conclusions:**

The application of the CO procedure in patients with LAR who were not indicated for stoma creation may contribute to reducing the risk of AL.

**Supplementary Information:**

The online version contains supplementary material available at 10.1007/s10151-025-03207-2.

## Introduction

Anastomotic leakage (AL) following rectal stapled anastomosis in rectal cancer surgery remains a serious complication, with reported incidence rates ranging from 3.5% to 18.3% [1–4]. AL is associated with increased morbidity, mortality, local recurrence, and compromised long-term functional outcomes [5–8]. Known risk factors for AL include male sex, tumor location, advanced tumor stage, perioperative bleeding, and neoadjuvant chemoradiotherapy [9–11]. In clinical practice, the decision to create a protective stoma is often based on the presence of high-risk factors. In the absence of these factors, surgeons may opt to perform low anterior resection (LAR) without fecal diversion. Nevertheless, AL can still occur in low-risk patients when a protective stoma is not created, with studies reporting leak rates between 11.3% and 13.6% in non-diverted LAR cases [12–14].

In gastrointestinal surgery, various attempts have been made to reduce AL by reinforcing stapled anastomoses, and these interventions have demonstrated the potential to mitigate the risk of AL [15–18]. Specifically, in rectal cancer surgery, several techniques have been explored. In laparoscopic LAR, intracorporeal suturing is performed at the intersection of the linear and circular staples created using the double-stapling technique [13]. Other studies have applied continuous suturing at this intersection and along the anterior aspect of the anastomosis [12, 19]. These studies demonstrated that staple line reinforcement can reduce the AL rate by over 50% compared to patients without reinforcement [12, 13, 19, 20]. Another study reported on performing transanal reinforcing sutures to reduce the need for stoma diversion [21].

However, staple line reinforcement with oversewing in rectal cancer surgery is challenging because of the narrow and deep pelvis, which makes both open and laparoscopic approaches challenging. Conversely, a robotic system, with its stable camera and enhanced dexterity, facilitates suturing in confined spaces [22–24]. Moreover, suturing the posterior aspect of the anastomosis, which is prone to dehiscence, is particularly challenging [25]. Some studies have only reinforced the intersection of the staple lines or the anterior aspect of the anastomosis; however, the use of a robotic system allows for relatively easier suturing of the posterior aspect.

In low-risk patients in whom a stoma is not indicated, a need to improve preventive strategies to minimize AL persists. Therefore, we hypothesize that circumferential oversewing (CO), where a circular staple line is encircled with barbed sutures to reinforce the stapled anastomosis, could reduce the incidence of AL in patients undergoing LAR without a diverting stoma. This study investigated whether CO in robotic LAR can effectively reduce the incidence of AL.

## Methods

### Study design and patients

We conducted a retrospective analysis of patients who underwent robotic LAR without fecal diversion at two tertiary centers between January 2015 and June 2023. The study included patients histologically diagnosed with rectal adenocarcinoma, excluding those who underwent the creation of a diverting stoma, received neoadjuvant chemoradiotherapy, or underwent concurrent major organ resections, such as hepatectomy or pneumonectomy. Since January 2019, the two centers have routinely performed CO procedures in LAR without fecal diversion. We compared patients who did not undergo CO (non-CO group) before 2019 with those who underwent CO (CO group) after 2019. Their baseline characteristics, including age, sex, American Society of Anesthesiologists physical status (ASA) score, body mass index (BMI), and tumor location from the anal verge (cm); operative data including operation time, transfusion, combined operation, number of stapler firings, and the use of CO; histopathologic findings of tumor size, T stage, and N stage; and postoperative data of anastomosis-related complications, overall complications, readmission, and reoperation were collected. Postoperative complications were assessed within the first 30 days after surgery. The current study protocol was approved by the institutional review board (IRB no. 2023-0222) and followed the standards outlined in the STROCSS 2021 criteria for reporting cohort studies on surgery [26].

### Surgical procedures and CO

**Fig. 1:**
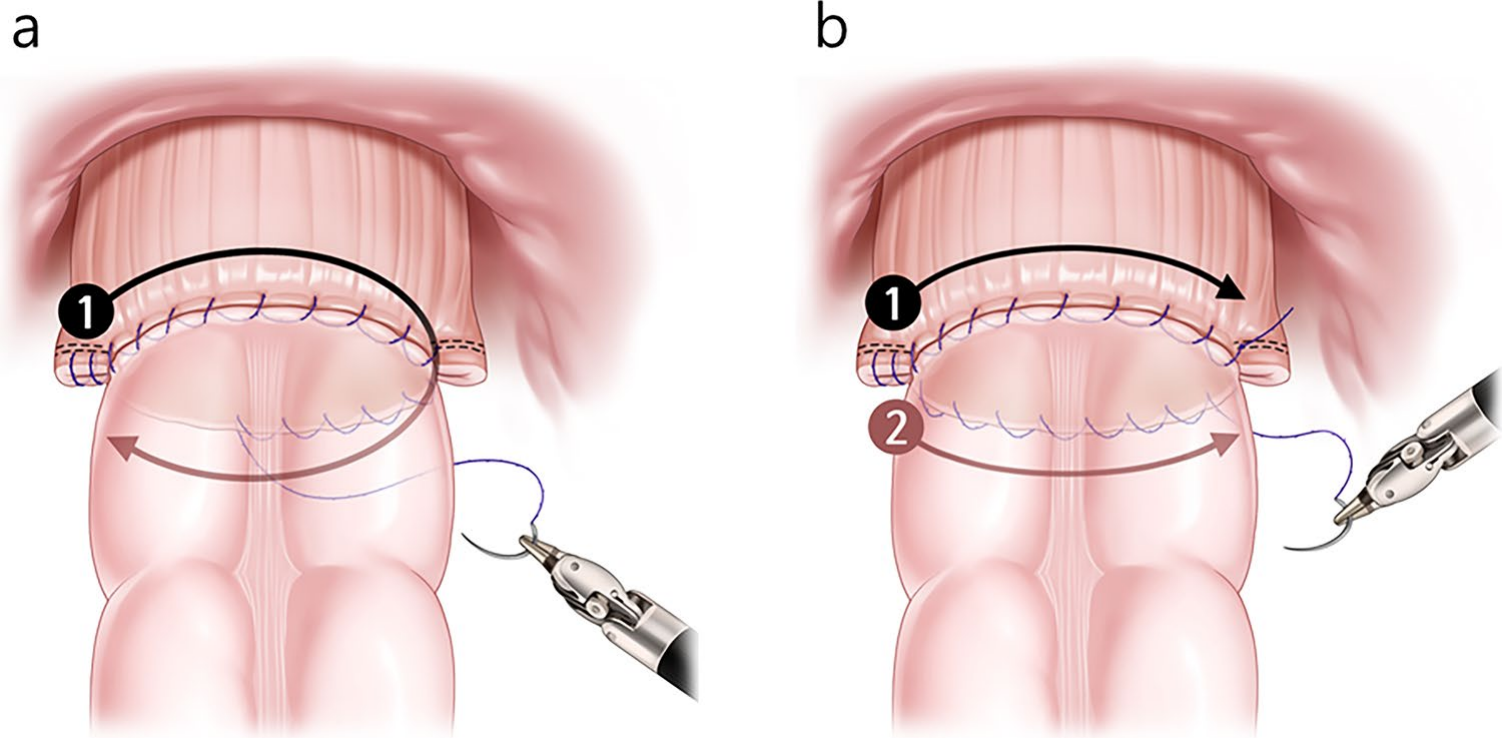
Schematic of circumferential oversewing. **a** Suturing with one suture material. **b** Suturing with two suture materials, each suturing at 180°

**Fig. 2:**
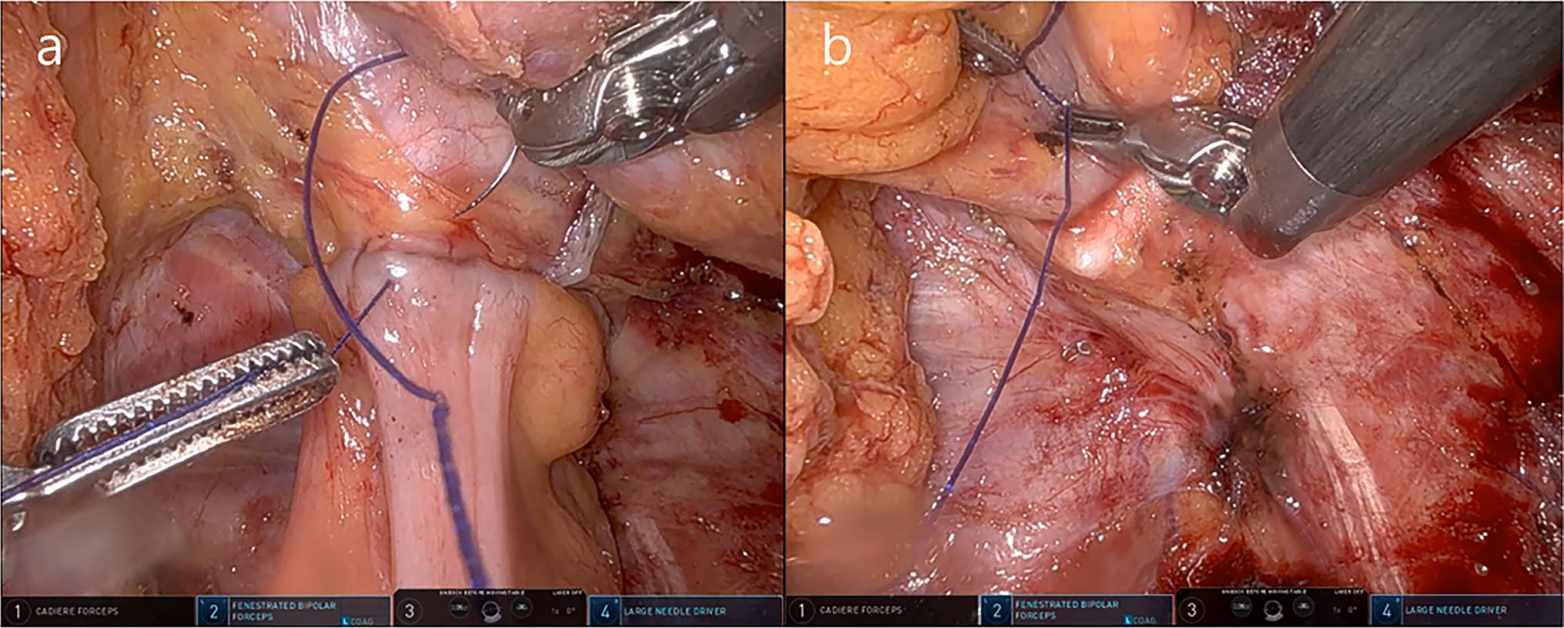
Intraoperative view of circumferential oversewing. **a** Anterior and **b** Posterior aspect of the anastomosis

Patients underwent mechanical bowel preparation, and prophylactic antibiotics were administered before incision and discontinued within 24 h post-surgery. The CO procedure was performed by two colorectal surgeons (Yang S and Yoon YS) using a da Vinci robotic system (Intuitive Surgical Inc., Sunnyvale, CA, USA). Robotic LAR included high or low ligation of the inferior mesenteric artery, total or tumor-specific mesorectal excision, and double-stapled rectal anastomosis. Decisions regarding whether to create a diverting stoma were made by each surgeon during the surgery, considering the risk factors and operation status. In the CO group, a full-thickness intracorporeal running suture with 3–0 barbed sutures, either V-LOC (Medtronic, Minneapolis, MN, USA) or Monofix (Hanmi Science, Seoul, South Korea) was applied circumferentially along the staple line, covering 360°. CO was performed using one of two methods. In the first method, suture material was used to suture the entire 360° (Fig. 1a). In the second method, two suture materials were used: one to suture the anterior of the anastomosis at 180°, and the other to suture the posterior at 180° (Fig. 1b). Figure 2 shows clinical photographs of the CO procedure, and the procedure is presented in the supplementary video material (Video 1). The mean time for CO was 20 min, based on the reviewed surgical recordings of 18 patients. The non-CO group received either reinforcement at staple line corners or no reinforcement. Splenic flexure mobilization was selectively performed for insufficient bowel length. An air-leak test was conducted, and a Jackson-Pratt drain was inserted in the pelvic cavity.

### Definition of AL

AL was defined as the presence of clinical signs indicating peritonitis (such as fever, elevated inflammatory markers, and signs of peritoneal irritation) alongside the drainage of feces through a drain or the identification of abnormal fluid collection in the pelvis on a computed tomography scan. AL was assessed within the first 30 postoperative days.

### Statistical analysis and inverse probability of treatment weighting (IPTW)

Categorical variables were reported as frequencies (%) and compared using Fisher’s exact or chi-square tests. Continuous variables were presented as mean ± standard deviation and compared using Student’s *t* test. Statistical analyses were conducted using the Statistical Package for the Social Sciences, version 24.0, for Windows (SPSS, IBM Corp., Armonk, NY, United States), and a *p* value < 0.05 was considered statistically significant. If propensity score matching is used for adjustment, it can lead to a reduction in sample size, subsequently resulting in diminished statistical power. Thus, in this study, we employed IPTW to mitigate the risk of data loss. IPTW based on propensity score modeling was used to account for the differences in baseline characteristics between the two groups. Baseline covariates potentially associated with AL, including sex, age, ASA score, BMI, and tumor location, were used to generate the underlying propensity score. Covariate balance was evaluated using the standardized mean difference, with a difference of < 10% considered acceptable. After adjusting for the IPTW method, the outcomes were analyzed, and the influence of CO on the AL, overall postoperative complication, and readmission rates were evaluated using a logistic regression model.

## Results

**Fig. 3:**
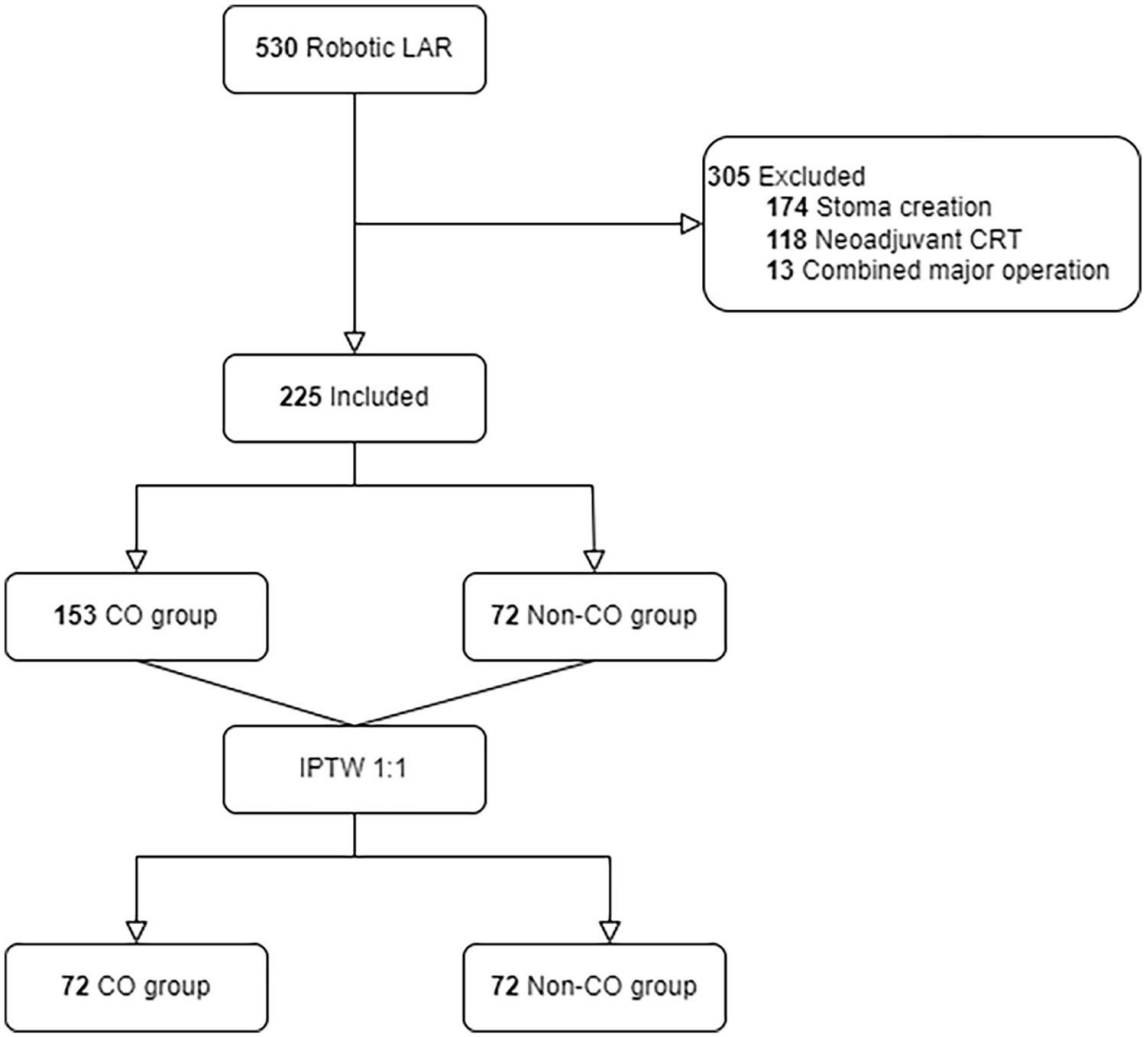
Flow diagram

A total of 530 patients who underwent robotic LAR were included in the study. Of these, 305 were excluded, including 118 who received neoadjuvant chemoradiotherapy, 174 with diverting stomas, and 13 who underwent major concurrent operations. Finally, 225 patients (153 with and 72 without CO) were analyzed (Fig. 3).

Considering that the CO group had different baseline characteristics, including a lower BMI than the non-CO group, a propensity score was generated using variables such as age, sex, BMI, ASA score, and tumor location. Baseline characteristics before and after IPTW are presented in Table 1. In the IPTW-adjusted patients, operative time was significantly longer in the CO group than in the non-CO group (198.7 ± 53.1 min vs. 180.1 ± 43.4 min, *p* = 0.010, Table 2). No significant differences were observed in operative parameters or pathological findings.

**Table 1:** Baseline characteristics of the patients before and after adjusting with IPTW

	Before IPTW			After IPTW		
	Circumferential oversewing					
	No (*n* = 72)	Yes (*n* = 153)	*p* value	No (*n* = 72)	Yes (*n* = 153)	SMD
Age, mean (SD), years	57.7 (9.6)	59.2 (10.5)	0.300	58.5 (9.2)	58.7 (10.4)	0.016
Sex (male), no. (%)	49 (68.1)	89 (58.2)	0.156	44 (61.5)	94 (61.4)	0.001
BMI, median (IQR), kg/m^2^	24.6 (22.7–26.1)	23.7 (22.0–25.1)	0.013	24.4 (22.4–25.5)	23.9 (22.2–25.4)	0.078
ASA score, no. (%)			0.724			0.038
1–2	66 (91.7)	138 (90.2)		66.1 (91.7)	138.8 (90.7)	
3–5	6 (8.3)	15 (9.8)		6.0 (6.2)	14.0 (9.3)	
Tumor location (from anal verge), mean (SD), cm	8.8 (2.6)	8.3 (1.9)	0.122	8 (2.4)	8 (1.9)	0.036

*ASA* American Society of Anesthesiologists, *BMI* body mass index, *IPTW* inverse probability of treatment weighting, *SMD* standardized mean difference, *SD* standard deviation, *IQR* interquartile range

**Table 2:** Surgical outcomes and pathologic results after adjusting with IPTW

	Circumferential oversewing		
	No (*n* = 72)	Yes (*n* = 153)	*p* value
Operation time, mean (SD), mins	180.1 (43.4)	198.7 (53.1)	0.010
Transfusion (yes), no. (%)	0 (0.0)	1 (0.7)	0.170
Number of stapler firings, no. (%)			0.259
≥ 3	2 (3.4)	2 (1.1)	
< 3	70 (98.6)	151 (98.9)	
Tumor size, mean (SD), cm	3.9 (2.2)	3.5 (2.2)	0.264
Pathologic T stage, no. (%)			0.493
T0–2	34 (47.6)	80 (52.5)	
T3–4	38 (52.4)	73 (47.5)	
Pathologic N stage, no. (%)			0.190
N0	41 (56.5)	100 (65.6)	
N+	31 (43.5)	53 (34.4)	

*SD* standard deviation

For postoperative outcomes, the overall complication rate was 12.0%, and the rate of AL was 4.5%. While no difference in the overall complications was observed between the two groups, patients with CO had a significantly lower AL rate than those without (1.7% vs. 10.3%, *p* = 0.010, Table 3). Considering that all patients with AL underwent reoperation, the reoperation rate was lower in the CO group than in the non-CO group (1.96% vs. 8.33%,* p* = 0.032). To evaluate the influence of CO on AL, overall complications, and readmission rates, a logistic regression model was used. The CO procedure was found to be a protective factor against AL (IPTW-adjusted OR 0.153, 95% CI 0.036–0.643; *p* = 0.010, Table 4).

**Table 3:** Postoperative complications after adjusting with IPTW

	Circumferential oversewing		
	No (*n* = 72)	Yes (*n* = 153)	*p* value
Overall complications	12 (16.7)	14 (9.2)	0.100
Anastomotic related complications			
Leakage	7 (10.3)	3 (1.7)	0.010
Bleeding	2 (2.2)	0 (0.0)	0.170
Stricture	0 (0.0)	1 (0.5)	0.919
Other complications			
Ileus	1 (1.1)	5 (3.2)	0.366
Pulmonary	1 (1.2)	1 (0.8)	0.744
Urinary	1 (1.8)	1 (0.9)	0.558
Others	1 (1.1)	4 (2.9)	0.419

**Table 4:** Analysis of AL, overall complications, and readmission using a logistic regression model before and after adjusting with IPTW

	Before IPTW			After IPTW		
	Circumferential oversewing					
	No	Yes	*p* value	No	Yes	*p* value
Anastomotic leakage	1	0.222 (0.053–0.906)	0.036	1	0.153 (0.036–0.643)	0.010
Overall complications	1	0.504 (0.220–1.153)	0.105	1	0.489 (0.216–1.103)	0.085
Readmission	1	0.700 (0.114–4.284)	0.700	1	0.797 (0.118–5.401)	0.817

*IPTW* inverse probability of treatment weighting

## Discussion

In this study, the AL rate was significantly lower in the group that underwent CO than in the group that did not undergo CO (1.7% vs. 10.3%; *p* = 0.010). AL can still occur in patients considered to be at low risk. Performing CO on these patients may significantly reduce the risk of AL. These findings are consistent with those of previous studies that demonstrated a reduction in AL with reinforcement sutures for rectal stapled anastomoses [12, 13, 20, 27]. However, to the best of our knowledge, this is the first study to specifically evaluate the effect of robotic CO applied to the entire 360° circumference of a rectal stapled anastomosis. Robotic CO may provide consistent reinforcement of the anastomotic line, potentially resulting in a reduced risk of leakage. These findings suggest that robotic CO may enhance anastomotic integrity and reduce postoperative complications, potentially leading to improved patient outcomes.

Studies that reinforced only the intersection of the staple lines or included reinforcement of the anterior aspect reported AL rates of approximately 2.4–4.8% in their reinforcing groups [12, 19]. In comparison, our study showed a relatively low AL rate of 1.7% in patients who underwent CO. This could be attributed to the effectiveness of CO. Meanwhile, the AL rate of 10.3% observed in the non-CO group in our study is comparable to that reported in other studies involving similar cohorts. In these cohorts, the AL rate for LAR without fecal diversion ranged from 10.5% to 13.6%, which is consistent with our findings [12, 13, 19].

In this study, we used a robotic surgical system to reinforce the entire circumference of the anastomosis with barbed sutures. In laparoscopic rectal surgery, reinforcement is typically focused on the vulnerable corners formed by the intersection of circular and linear staple lines, as studies have shown that reinforcing these areas can reduce the rate of AL [12, 13, 28, 29]. In previous Chinese and Japanese studies, only the anterior aspect of the staple line, including the intersections, was reinforced using barbed sutures [12, 19]. However, AL can occur beyond these intersections, with one study reporting that 81% of anastomotic defects were located posteriorly and posteriolaterally [25]. Thus, reinforcing the posterior aspect is also crucial. In our study, we reinforced not only the intersections but also the entire anastomotic circumference. Although suturing within the pelvis during laparoscopic surgery is challenging because of the limited range of motion and the need for careful manipulation, the robotic system makes this achievable [30–33]. The articulated robot arm provides freedom to control needle entry and exit without excessive bowel movement, facilitating consistent reinforcement of the posterior aspect of the anastomosis.

Although the proportion of male patients was higher, our cohort primarily consisted of patients expected to have a lower risk of AL, characterized by low ASA scores and BMI. The preponderance of lower ASA scores in our study can be attributed to the likelihood that patients with higher ASA scores are more prone to stoma creation; thus, these patients were excluded from the analysis. Furthermore, the observed lower BMI is consistent with the findings of other studies conducted in Asian populations, which typically report lower BMI values than Western cohorts [12, 21]. In our practice, stomas are created for patients with certain risk factors, including male sex, comorbidities, obesity, perioperative bleeding, ultra LAR, and neoadjuvant chemoradiotherapy [34–36]. Patients who received a stoma owing to these risk factors were excluded. We confirmed that CO was effective in low-risk patients; therefore, caution is required when interpreting these results. However, in one study, patients with one or more risk factors were classified as high risk, and the use of reinforcing sutures even in the high-risk group was shown to be effective in reducing the incidence of AL [13]. In addition, a randomized controlled trial compared LAR with transanal reinforcement with LAR with protective ileostomy. The results showed similar AL rates between the two groups. This study demonstrated that staple line reinforcement can be as effective as protective stoma reinforcement in reducing the incidence of AL [37]. Therefore, further studies on the effectiveness of CO are required. Specifically, future studies should investigate whether CO is effective in patients with high-risk factors, or whether CO can be as effective as stoma creation in preventing AL.

Our study cohort had relatively lower BMI, which is similar to previous studies on staple line reinforcement in rectal cancer surgery. Most of these studies have been performed in Asian populations [27]. Studies examining reinforcement techniques in Western populations with higher BMI are limited. However, evidence from bariatric surgery shows that staple line reinforcement effectively reduces leak rates in patients with high BMI [16, 17]. This indicates that the benefits of reinforcement may not differ significantly according to BMI. While high BMI can create technical difficulties during rectal cancer surgery, our study included patients with various BMI—28% of patients in the oversewing group had BMI ≥ 25 kg/m^2^, with some patients exceeding 36 kg/m^2^. Three patients who developed anastomotic leakage had BMI ≤ 23 kg/m^2^, while no leaks occurred in higher BMI patients who received circumferential oversewing. This demonstrates that the procedure can be performed safely and may be effective even in patients with higher BMI. Future studies focusing on Western populations with higher BMI would help validate these findings.

Meanwhile, several issues are associated with robotic CO procedures. One significant limitation of the robotic system is the absence of tactile feedback, which means that the surgeon must rely entirely on visual cues to judge the tension during suturing. This lack of haptic perception could lead to excessive force being applied to the anastomosis, increasing the risk of ischemia at the anastomotic site, and potentially contributing to AL. Despite this limitation, the robotic system provided precise maneuverability in confined spaces, thereby reducing the need for excessive tissue manipulation. However, if the bowel condition is unfavorable owing to radiation therapy-induced enteritis or the presence of bulky tumors, AL may occur even if the CO procedure was meticulous. Under these conditions, splenic flexure mobilization is necessary to secure a healthier proximal bowel, and the creation of a diverting stoma is recommended.

The present study has several limitations. First, our study had a relatively small sample size of 72 patients. To address this limitation, we employed IPTW rather than propensity score matching to balance baseline characteristics and reduce selection bias while preserving the entire dataset and avoiding further data loss. Second, this was a retrospective, nonrandomized observational analysis focusing on low-risk patients who did not receive neoadjuvant chemoradiotherapy, underwent concurrent major organ resection, or required stoma creation. Despite this limitation, the study provides meaningful insights into the potential benefits of CO for reducing AL in patients not indicated for stoma creation and with an expected low risk for AL. Third, CO was performed in the latter part of the study period, raising the possibility that improved outcomes may be due to increased surgeon experience. However, it should be noted that both surgeons had already performed over 500 minimally invasive rectal cancer surgeries in 2015 and had accumulated more than 100 cases of robotic rectal surgeries since the early 2010s. Therefore, it seems more likely that the improvement in AL rates was influenced by the CO procedure, rather than merely by the improved experience of the surgeon over time. Finally, the small number of AL events limited the statistical power of the study. Despite conducting a multicenter study to increase sample size, AL was confirmed in only 10 patients, which may have contributed to the low statistical power. To further validate the effectiveness of the CO procedure and address these limitations, large-scale randomized prospective studies are necessary.

In conclusion, the application of the CO procedure in patients with LAR who are not indicated for stoma creation may provide structural support to the stapled rectal anastomosis and contribute to a reduction in the risk of AL. To further elucidate the generalizability and efficacy of CO, future randomized, prospective studies with larger, multicenter sample sizes including international centers with Western patients are necessary. Such studies would provide more robust evidence and broaden the applicability of our findings.

## Supplementary Information

Below is the link to the electronic supplementary material.

Supplementary file1 (MP4 282147 KB)

## Data Availability

The datasets examined in the current study can be obtained from the corresponding author upon reasonable request.
